# ZNF93 Increases Resistance to ET-743 (Trabectedin; Yondelis®) and PM00104 (Zalypsis®) in Human Cancer Cell Lines

**DOI:** 10.1371/journal.pone.0006967

**Published:** 2009-09-09

**Authors:** Zhenfeng Duan, Edwin Choy, David Harmon, Cao Yang, Keinosuke Ryu, Joseph Schwab, Henry Mankin, Francis J. Hornicek

**Affiliations:** 1 Sarcoma Biology Laboratory, Center for Sarcoma and Connective Tissue Oncology, Massachusetts General Hospital, Boston, Massachusetts, United States of America; 2 Cancer Center/Hematology Oncology, Massachusetts General Hospital, Boston, Massachusetts, United States of America; Dresden University of Technology, Germany

## Abstract

**Background:**

ET-743 (trabectedin, Yondelis®) and PM00104 (Zalypsis®) are marine derived compounds that have antitumor activity. ET-743 and PM00104 exposure over sustained periods of treatment will result in the development of drug resistance, but the mechanisms which lead to resistance are not yet understood.

**Methodology/Principal Findings:**

Human chondrosarcoma cell lines resistant to ET-743 (CS-1/ER) or PM00104 (CS-1/PR) were established in this study. The CS-1/ER and CS-1/PR exhibited cross resistance to cisplatin and methotrexate but not to doxorubicin. Human Affymetrix Gene Chip arrays were used to examine relative gene expression in these cell lines. We found that a large number of genes have altered expression levels in CS-1/ER and CS-1/PR when compared to the parental cell line. 595 CS-1/ER and 498 CS-1/PR genes were identified as overexpressing; 856 CS-1/ER and 874 CS-1/PR transcripts were identified as underexpressing. Three zinc finger protein (ZNF) genes were on the top 10 overexpressed genes list. These genes have not been previously associated with drug resistance in tumor cells. Differential expressions of ZNF93 and ZNF43 genes were confirmed in both CS-1/ER and CS-1/PR resistant cell lines by real-time RT-PCR. ZNF93 was overexpressed in two ET-743 resistant Ewing sarcoma cell lines as well as in a cisplatin resistant ovarian cancer cell line, but was not overexpressed in paclitaxel resistant cell lines. ZNF93 knockdown by siRNA in CS-1/ER and CS-1/PR caused increased sensitivity for ET-743, PM00104, and cisplatin. Furthermore, ZNF93 transfected CS-1 cells are relatively resistant to ET-743, PM00104 and cisplatin.

**Conclusions/Significance:**

This study suggests that zinc finger proteins, and ZNF93 in particular, are involved in resistance to ET-743 and PM00104.

## Introduction

ET-743 (Yondelis®; Trabectedin) is marine alkaloid derivative isolated from the Caribbean sea squirt *Ecteinascidia turbinate* [1], [2]. ET-743 has a broad spectrum of activity in tumor cell lines at pM and low nM concentrations, and it also has clinical activity towards ovarian cancer, breast cancer, and sarcomas [2], [3]. ET-743 has been approved by the EMEA/EU for patients with advanced sarcomas who have either progressed after treatment with an anthracycline or are not clinically suitable to receive conventional agents [4]. ET-743 is composed of three tetrahydroisoquinoline subunits containing a central carbinolamine moiety enabling it to covalently bind to DNA. ET-743 binds to the minor groove of the DNA helix with sequence-specific binding preference for GC-rich triplets and subsequently forms covalent adducts with the N2-position of guanine. As a result, the minor groove is exposed and turned toward the major groove. When such binding occurs, DNA strands become cross-linked and cannot be replicated, resulting in cell death [5], [6]. The direction of DNA turning is a novel feature among DNA minor groove-interactive agents, thus making ET-743 unique.

PM00104 (Zalypsis®), derived from mollusks, is a novel chemical entity related to Jorumycin, a marine natural compound belonging to the family of *Renieramycins*, obtained from sponges [7], [8]. PM00104 has *in vitro* and *in vivo* anti-tumor activity towards a wide variety of solid and hematological tumors. As with ET-743, PM00104 also binds to DNA and is cytotoxic; however, unlike ET-743, PM00104 does not activate the “DNA damage checkpoint” response. Thus, the cytotoxic effects of PM00104, although dependent on DNA binding, are not triggered by DNA damage response mechanisms[8].

As with other chemotherapeutic drugs, ET-743 and PM00104 exposure over sustained periods of treatment will result in the development of drug resistance, but the mechanisms are not well understood. Different studies have reported conflicting descriptions of the relationship between ABCB1 expression and ET-743 resistance in human cancer cell lines [9], [10]. In the human ovarian cell line IGROV-1, which is selected for ET-743 resistance, ABCB1 is overexpressed [11]. Sensitivity could be restored by the addition of the Pgp1 inhibitor PSC-833, suggesting that ET-743 may be a Pgp1 substrate. Another study however, observed that two Pgp over-expressing human epidermoid cancer cell lines (KB-8-5 and KB-C-2) were not resistant to ET-743 [9]. Notably, sub-lethal concentrations of ET-743 could reverse resistance to doxorubicin and vincristine in these cell lines. There are no reports describing the mechanism of PM00104 resistance in tumor cells.

Chondrosarcomas are a heterogeneous group of tumors of mesenchymal origin that develop in bone or cartilage and display chondrocytic characteristics[12]. These tumors respond poorly to conventional treatments such as chemotherapy and radiation, and prognosis is related to tumor grade and differentiation status. The sensitivity of sarcoma cells to ET-743 and PM00104 has led us and others to consider these compounds as interesting candidates for future treatment of chondrosarcoma [13], [14], [15]. However, as with other chemotherapeutic agents, the propensity of tumor cells to develop resistance to ET-743 or PM00104 poses a significant challenge for using this drug over an extended period of time. The goal of this study is to explore the mechanisms of ET-743 or PM00104 resistance in chondrosarcoma.

## Material and Methods

### Chemotherapy drugs

ET-743 (Yondelis®; Trabectedin) and PM00104 (Zalypsis®) were supplied by PharmaMar (Spain). Paclitaxel (Taxol®), Doxorubicin (Adriamycin RDF®, EC No. 2468183), Methotrexate (Trexall) and Cisplatin (Cisplatinum; CDDP) were obtained through unused residual clinical material provided by the pharmacy at the Massachusetts General Hospital. The stock solution of drugs were prepared according to the drug specifications and stored at −20°C.

### Chondrosarcoma cell line CS-1

The human chonodrosarcoma cell line, propagated since 1999 and designated CS-1, were established from a surgically resected human high grade chonodrosarcoma that was not previously exposed to chemotherapy or radiation therapy, and grown in monolayer. Briefly, tissue was aseptically obtained immediately following resection, placed in RPMI-1640 tissue culture medium supplemented with 10% fetal bovine serum, and the tissue cultured at 37°C incubator containing 5% CO2 [13], [14]. The detailed analysis of CS-1 has been reported previously [13], [14], [15], [16], [17]. Total RNA was extracted after CS-1 was passaged four times.

### Establishment of ET-743 or PM00104 resistant cell line

CS-1/ER and CS-1/PR cells resistant to ET-743 or PM00104 were established from the CS-1 parent cell line by exposure to ET-743 or PM00104 with step-by step increase concentration of ET-743 or PM00104 for one year using similar method as previously reported [18], [19], [20]. In brief, cultured CS-1 cells were exposed to 0.00001 µM either ET-743 or PM00104 for 7 days and then washed and cultured in medium containing 0.00003 µM for 7 days. If the CS-1 cells demonstrated cytotoxicities after exposure to a given concentration of ET-743 or PM00104, they were washed and cultured in drug-free medium for 7 days. For example, we noticed 90% of CS-1 cells were killed when cells were exposed to 0.001 µM of ET-743 or 0.003 µM of PM00104 for 7 days. When the viable cells had recovered, they were seeded in medium containing the most recently exposed concentration of drug again for 3 days. After repeated several cycles and when the cells grew up to 70% confluence in the culture medium containing drugs, the concentration of drugs will increased to the next level. After one year, the ET-743 resistant cell line CS-1/ER and PM00104 resistant cell line CS-1/PR were established as determined by MTT assay. These cells represent populations of drug resistant cells rather than a selected cloned population. CS-1/ER can grow in concentrations of 0.005 µM ET-743 and CS-1/PR can grow up in the concentration of 0.01 µM PM00104.

### Other drug resistant cell line used in this study

Human ovarian cancer cell lines SKOV-3 and A2780 and a human breast cancer cell line MCF-7 were purchased from American Type Tissue Collection (Rockville, MD). The paclitaxel-resistant SKOV-3_TR_, MCF-7_TR_ and cisplatin-resistant A2780cp cell lines were established as previously reported [19], [20]. Dr. Katia. Scotlandi (Institute Orthopedics Rizzoli, Italy) provided Ewing sarcoma ET-743 resistant cell line TC-ET [21].

### Tissue culture

All the cell lines were cultured in RPMI 1640 (Invitrogen, Carlsbad, CA) supplemented with 10% fetal bovine serum, 100-units/ml penicillin and 100 µg/ml streptomycin (Invitrogen). Cells were incubated at 37°C in 5% CO_2_-95% air atmosphere and passaged when near confluent monolayers were achieved using trypsin-EDTA solution. Drug-resistant cell lines were periodically cultured in the respective drug to confirm their drug resistance characteristics. Cells were free on mycoplasma contamination as tested by MycoAlert(R) Mycoplasma Detection Kit from Cambrex (Rockland, ME).

### Cytotoxicity assay

Drug cytotoxicity was assessed in vitro using the MTT assay as previously described [22]. Briefly, 2×10^3^ cells per well were plated in 96-well plates in culture medium (RPMI 1640 supplemented with 10% fetal bovine serum and penicillin/streptomycin) containing increasing concentrations of drug. After 7 days of culture, 10 µl MTT (5 mg/ml in PBS, obtained from Sigma) were added to each well and the plates were incubated for 4 h. The resulting formazan product was dissolved with acid-isopropanol and the absorbance at a wavelength of 490 nm (A_490_) was read on a SPECTRAmax® Microplate Spectrophotometer (Molecular Devices, Sunnyvale, CA). The absorbance values were normalized by assigning the value of the control line in the medium without drug to 1.0 and the value of the no cell control to 0. Experiments were performed in triplicate.

### RNA extraction

Total RNA was collected from CS-1 (parental cell line with passaged 4 times) , CS-1/ER and CS-1/PR ( resistant daughter cell lines with cultured for one year) using TRIzol® Reagent (GIBCO, Grand Island, NY) according to the manufacturer's instructions. To account for and eliminate biologic noise, RNA was isolated from three distinct flasks of each cell line. These biologic replicates were pooled. RNA quality was determined *via* ethidium bromide staining following agarose/formaldehyde gel electrophoresis.

### Gene transcriptional profiling

Total RNA was processed and hybridized to Affymetrix Gene Chip U133 Plus 2.0 arrays (Santa Clara, CA) by the Gene Array Technology Center at Harvard Medical School (http://genome.med.harvard.edu). Affymetrix Gene Chip U133 Plus 2.0 in the first and most comprehensive whole human genome expression array. This array completes coverage of the whole human Genome with over 47,000 transcripts. Each probe consists of 20 separate 23-mer oligonucleotides. The expression level of each mRNA is quantified by measuring its hybridization to these 23-mers in comparison to its hybridization to a one-base mismatch oligonucleotide.

### Data analysis

GeneSifter was used to analyze the microarray data (http://www.genesifter.net/web/). GeneSifter provides powerful analytical algorithms (RMA, PAM, ANOVA, CLARA, Benjamini-Hochberg, etc.) through an intuitive web interface. GeneSifter can identify differentially expressed genes and cluster analysis for identifying patterns of gene expression and segregating genes based on these patterns. Fold change in expression between sensitive and resistant cell lines was evaluated using the Mann-Whitney test. A tenfold or greater change in intensity combined with a Mann-Whitney associated P value less than 0.05 was used as the criterion for inclusion in our filtered data set. Intensity information was exported to Microsoft Excel as needed.

### TaqMan reverse transcription-PCR for quantification of differentially expressed gene

Real-time RT-PCR were performed to validate differentially expressed genes. For gene expression detection, cDNA reverse transcription was performed from total RNA samples using specific oligo dT primers from the Taq Man RNA Assays and reagents from the TaqMan RNA Reverse Transcription kit (Applied Biosystems). The resulting cDNA was amplified by PCR using Taq Man Zinc finger protein 93 (ZNF93), ZNF43 and THADA gene assay primers with the Taq Man Universal PCR Master Mix and analyzed with a StepOnePlus Real time PCR System according to the manufacturer's instructions (Applied Biosystems). GAPDH and actin were used as a control. The relative levels of gene expression were calculated from the relevant signals by normalization with the signal for GAPDH or actin expression. PCR reaction mixtures contained Taq Man human ZNF93, or ZNF43 and THADA and Universal PCR Master Mix in a total volume of 20 AL. Cycling variables were as follows: 95°C for 10 min followed by 40 cycles at 95°C (15 s) and annealing/extension at 60°C (1 min). All reactions were performed in triplicate.

### ZNF93 siRNA assay

For ZNF93 (GenBank accession number: NM_031218) interference, sense ZNF93 oligo (5′-CCUCUACCCUUAGUUCACAtt-3′) and antisense ZNF93 oligo (5′-UGUGAACUAAGGGUAGAGGag-3′) were purchased from Applied Biosystems and was used according to the manufacturer's instructions. For validation of ZNF93 RNAi specificity, the siGenome SMARTpool Human ZNF93 siRNAs were purchased from Dharmacon (Chicago,IL) with the following four target sequences of ZNF93 gene. Target sequence 1: 5′-GGACUUAACCAGUGUAGUA-3′; target sequence 2: 5′-AACCAAUCCUCGACACUUA-3′;target sequence 3: 5′-GCCCUACGUUUGUGAAGAA-3′ and target sequence 4: 5′-CCUUAUAGAUGUAGAGAAU-3′. For transfection, cells were either plated on 96 well plates for MTT assays or plated on dishes for RNA extraction. Transfections were performed with siPORT™ *NeoFX*™ siRNA transfection reagents (Ambion. Inc, Austin, TX) as directed by the manufacturer. The Silencer™ EGFP siRNA (Ambion) and siRNA Control® reagent (Dharmacon) were used as positive and negative controls in all experiments. For ZNF93 inhibition, the final concentration of siRNA was either at 25 nM or 100 nM. Media was replaced with RPMI1640 supplemented with 10% FBS 24 hours after transfection. Total RNA was isolated after 72 hours of ZNF93 siRNA transfection for Real-time RT-PCR confirmation.

### pIRES_ZNF93_ expression vector construction and transfection

A 1907 base pair cDNA fragment containing the full ORF of human ZNF93 was amplified by RT-PCR from the RNA of CS-1/PR cell line which highly overexpresses ZNF93. RT-PCR was performed using the sense and antisense primers to human ZNF93: sense primer 5′-ATAAGAATGCGGCCCGGAAGCCTAGAAATGGGACCATTG-3′ to introduce a Not I site as underlined, and antisense primer: 5′-GGTGGATCCTCACACTTCTAGGGTTTCT-3′ ( GenBank # NM_031218 ) to introduce a BamH I site as underlined. The introduced restriction enzyme sites were designed for following transfection studies. Clonetech's (Palo Atlo, CA) mammalian expression vector pIRESneo was used for functional expression study. The resulting ZNF93 RT-PCR product was cloned into pCR®2.1 vector using Invitrogen's Original TA Cloning Kit. After sequence confirmation, ZNF93 was cut from the pCR®2.1 vector, purified, subcloned into the MCS of expression vector pIRESneo, and subsequently sequenced to confirm the correct ORF. Expression of ZNF93 cDNA was under the control of the pCMV. Transfections were performed using LipofectAmine Plus reagents (Invitrogen) as follows: approximately 5×10^5^ CS-1 cells were plated into 90 millimeter tissue culture dishes and cultured overnight. Prior to transfection, the growth medium was replaced with serum free RPMI 1640 and cultured for three hours. LipofectAmine reagent containing 5 µg of pIRES_empty_, pIRES_ZNF93_ was combined with Plus reagent and applied to the cells. After culture for four hours, the media was replaced with RPMI 1640 containing 10% fetal bovine serum. G418 sulfate (Invitrogen) selection (300 µg/ml) was started at 24 hours post transfection. The selection medium was changed every 2 days. Effects of overexpression ZNF93 on ET-743 and PM00104 were determined by MTT cytotoxicity assay.

## Results

### CS-1/ER, CS-1/PR cell lines are resistant to several chemotherapeutic agents

**Figure 1:**
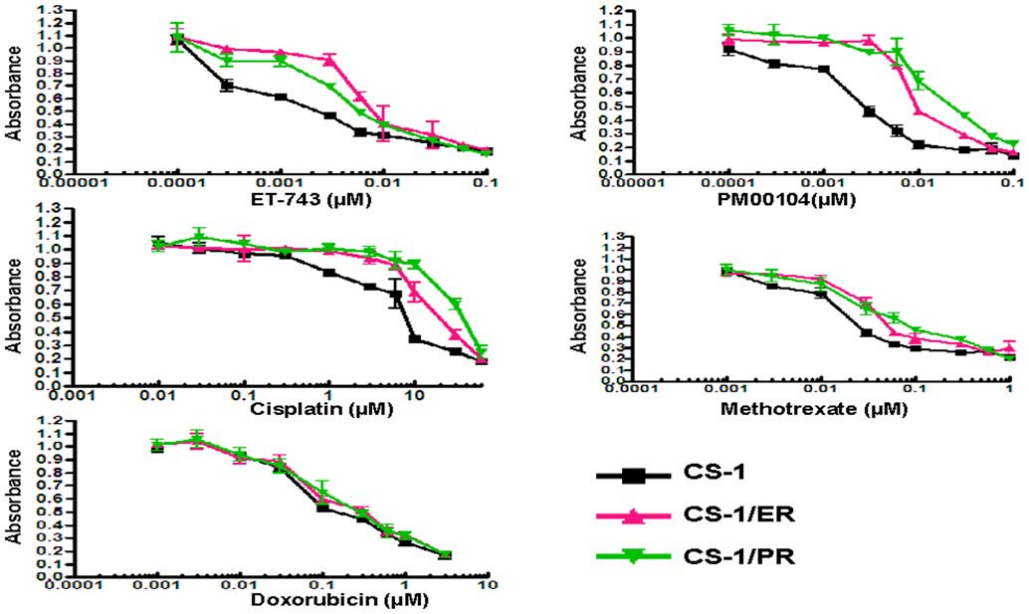
CS-1/ER and CS-1/PR drug sensitivities. CS-1/ER, CS-1/PR and CS-1 cells were exposed to varying concentrations of drugs for 6 days. Growth inhibition was determined by incubation with the tetrazolium dye MTT and by absorbance measurement at 490 nm. Data reflect three replicates at each concentration.

CS-1/ER and CS-1/PR were selected from the parental cell line, CS-1, by exposure to stepwise increases in ET-743 or PM00104 concentrations for a period of one year. The drug resistance phenotype was found to be stable after 14 months of continuous culture in drugs or at least 6 months under drug-free conditions. No significant difference between CS-1 and CS-1/ER or CS-1/PR cells was observed *in vitro* cell growth (similar doubling time) and microscopic morphology. MTT cytotoxicity analysis shows that CS-1/ER, CS-1/PR are 10- 20 fold more resistant to ET-743 and PM00104 as compared with the sensitive parent cell line. The IC_50_-value of ET-743 in CS-1 cells was 0. 0008 µM as compared to 0. 01 µM in CS-1/ER cells (12.5-fold resistance). The IC_50_-value of PM00104 in CS-1 cells was 0. 002 µM as compared to 0. 04 µM in CS-1/PR cells (20-fold resistance). In addition, CS-1/ER and CS-1/PR exhibits resistance to cisplatin and methotrexate but not to doxorubicin (Fig. 1).

### A large number of genes are associated with ET-743 and PM00104 resistance

**Figure 2:**
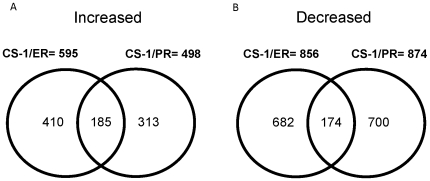
Venn diagram of genes overexpression (A), underexpression (B) in CS-1/ER, CS-1/PR as compared with CS-1. Genes over expressed/under expressed in more than one cell line are indicated in the overlapping regions of the circles.

Human Affymetrix Gene Chip U133 Plus 2.0 arrays were used to examine relative RNA expression levels between CS-1, CS-1/ER, and CS-1/PR. The expression profiles of these three chondrosarcoma cell lines were evaluated by Genesifter. In addition, we have submitted our microarray data to **G**ene **E**xpression **O**mnibus (http://www.ncbi.nlm.nih.gov/geo/) and these array data have been assigned a GEO accession number as GSE16748. We found a large number of genes had significantly different levels of expression in CS-1/ER and CS-1/PR as compared to CS-1. To focus on genes with only significant changes in expression levels, we identified genes with a ten-fold or greater change in expression levels. Using this criteria, 595 (CS-1/ER), 498 (CS-1/PR) genes exhibited more than ten-fold overexpression in the ET-743 and PM00104 resistant lines relative to their expression in the sensitive parental lines (Fig. 2A). In addition, 856 (CS-1/ER), 874 (CS-1/PR) transcripts were more than ten-fold decreased in the resistant cell lines as compared to controls (Fig. 2B). Changes in expression levels ranged from 10- to 536- fold. The genes identified were largely non-overlapping between cell lines and encoded for proteins with a wide variety of biochemical functions. The genes identified encode for proteins that bind DNA have catalytic activities, molecular transducer activities, regulate transcription, transport proteins and molecules, regulate enzymes and there are many other genes that have limited characterization. The top 20 most highly overexpressed and underexpressed genes in each of the resistant cell lines are summarized in Table 1. There were seven genes overexpressed in both CS-1/ER and CS-1/PR and one gene that underexpressed in both cell lines. In these two resistant cell lines, there are more over-lapping gene in the top 20 over expressed genes list as compared to the under expressed genes list. We also noticed the degree of change in gene expression was more dramatic in the set of under expressed genes (Table 1).

**Table 1:** A list of the 20 most differentially expressed genes in CS-1/ER and CS-1/PR as compared with CS-1.

CS-1/ER *vs* CS-1	CS-1/PR *vs* CS-1
**Overexpressed genes**	
Family with sequence similarity 9 (295)	Metastasis related protein MB2 (91)
***Zinc finger protein 93*** ** (268)**	Transcribed locus AW190406 (89)
** ** ***Thyroid adenoma associated*** ** (125)**	mRNA clone IMAGE:5229134 (81)
Transcribed locus AU156801 (120)	Gremlin2, cystein knot superfamily (62)
***Transcribed locus EMB gene*** ** (88)**	Translocase of inner mitochondrial (60)
***Coiled-coil domain containing 144B*** ** (87)**	***Zinc finger protein 43*** ** (56)**
Vitamin B12 binding protein (86)	ESTs AV724769 (56)
***ESTs AI655611*** ** (76)**	ESTs AV696977 (56)
***Transcribed locus AI8200854*** ** (75)**	***Coiled-coil domain containing 144B*** ** (55)**
cDNA clone ADKA02259 (74)	***Transcribed locus AI8200854*** ** (47)**
Melanoma antigen family A 2 MAGE A2 (69)	***Transcribed locus EMB gene*** ** (46)**
Zinc finger protein 568 (63)	***Zinc finger protein 93*** ** (45)**
Transcribed locus AW590062 (59)	ESTs AI057226 (44)
ESTs H14374 (58)	RAB7A, member RAS oncogene family (44)
Kinestin family member 17 (57)	***Thyroid adenoma associated*** ** (41)**
mRNA C19orf9 (57)	Serine/threonine kinase 32A (40)
Transcribed locus AA503877 (55)	Multiple EGF-like-domanis 10 (39)
***Zinc finger protein 43*** ** (55)**	mRNA clone IMAGE:4670253 (39)
ESTs AI061288 (53)	Dpy-19-like 2 (39)
Glutamate receptor AMPA4 (48)	***ESTs AI655611*** ** (39)**
**Underexpressed genes**	
Phosphoglucomutase 5 (470)	Sperm proein AF098306 (535)
Deleted in azoospermia 1 (366)	Sperm proein NM-013453 (412)
Biglycan AA845258 (319)	Lung type-1 cell membrane glycoprotein (127)
Transcribed locus BC015772 (313)	Deleted in axoospermina 2 (110)
Collagen, type III, alpha AI813758 (176)	cDNA clone IMAGE:4791911 (109)
mRNA clone FLC1492 (166)	Cartilage glycoprotein-39 (108)
ATP-binding cassette, sub-family G 1 (150)	ESTs AI758697 (94)
Biglycan NM_001711 (147)	Cancer/testis antigen 1A (88)
Cartilage gycoprotein-39 (139)	cDNA clone FLJ38810(83)
Collagen, type III, alpha AU144167 (134)	Cancer/testis antigen 2 (80)
Keltch domain containing 1 (124)	Transcribed locus AI651510 (78)
cDNA FLJ43080 (107)	cDNA FLJ11909 (73)
***Fibrillin 2*** ** (101)**	Cadherin 3, type 1 (66)
Odd Ox/ten-m homolog 2 (100)	Cancer/testis antigen 1A (72)
Transcribed locus AI384053 (87)	mRNA regulated in glioma (63)
Optineurin (86)	Regulatory factor X, 3 (61)
cDNA FLJ14388 (86)	***Fibrillin 2*** ** (58)**
cDNA clone RP4-76112 (74)	thymic stromal lymphopoietin (58)
Chemokine ligand 3 (71)	EPH receptor A4 (55)
Phosphoglucomutase 5 (70)	Sulfatase 2 (46)

Genes in bold type are differentially expressed in both two resistant cell lines. The number in parentheses following the gene name is the fold overexpression/underexpression compared to the respective sensitive cell line.

### Genes differentially expressed in both resistant cell lines

Fig. 2 Venn diagram shows that 185 genes were overexpressed, 174 genes were underexpressed in the both CS-1/ER, and CS-1/PR resistant cell lines in comparison to CS-1 (Fig. 2A and Fig. 2B). The top 20 most highly overexpresssed genes in both resistant cell lines are summarized in Table 2. As 3 out top 20 overecpressing genes in both CS-1/ER, and CS-1/PR resistant cell lines are zinc finger protein genes (ZNF93, ZNF43 and ZNF568), we decided to further validate these genes by real-time RT-PCR.

**Table 2:** Top 20 overexpressed genes (>10 fold) in both CS-1/ER and CS-1/PR resistant cell lines.

Gene name	GenBank accession no	Chromosome location	Function
Family with sequence similarity 9	AF494344	Xp22	unknown
**Zinc finger protein 93** *	BC020837	19p12	transcriptional regulation
**Thyroid adenoma associated**	BC037990	2p21	unknown
Coiled-coil domain containing 144B	AL162091	17p11	transcriptional regulation
Transcribed locus EMB Gene	BE080109	5q11	regulator of cell/ECM interactions
Clone IMAGE:5229134 mRNA	BC037976	13p11	unknown
Transcribed locus	AU156801	1p17	unknown
Transcribed locus	AI820854	14p16	unknown
ESTs	AI655611	1p11	unknown
**Zinc finger protein 43**	AK022905	19p13	transcriptional regulation
Metastasis related protein (MB2)	AF100640	unknown	unknown
Translocase of inner mitochondrial	BC005236	Xq22	chaperone-like protein
Transcribed locus	AW190406	7p7	unknown
Vitamin B12 binding protein	NM_001062	11q11	transports cobalamin into cells
ESTs	AV724769	3p8	unknown
Zinc finger protein 568	BC031218	19q13	transcriptional regulation
Clone ADKA02259	AK024520	2p18	unknown
Dpy-19-like 2	AA758751	7p14	unknown
Chromosome 19 ORF 9 (C19orf9)	NM_152659	unknown	unknown
Transcribed locus	AA503877	1p11	unknown

* Genes in bold were chosen for real-time RT-PCR validation.

### TaqMan real-time RT-PCR analysis of differentially expressed genes

**Figure 3:**
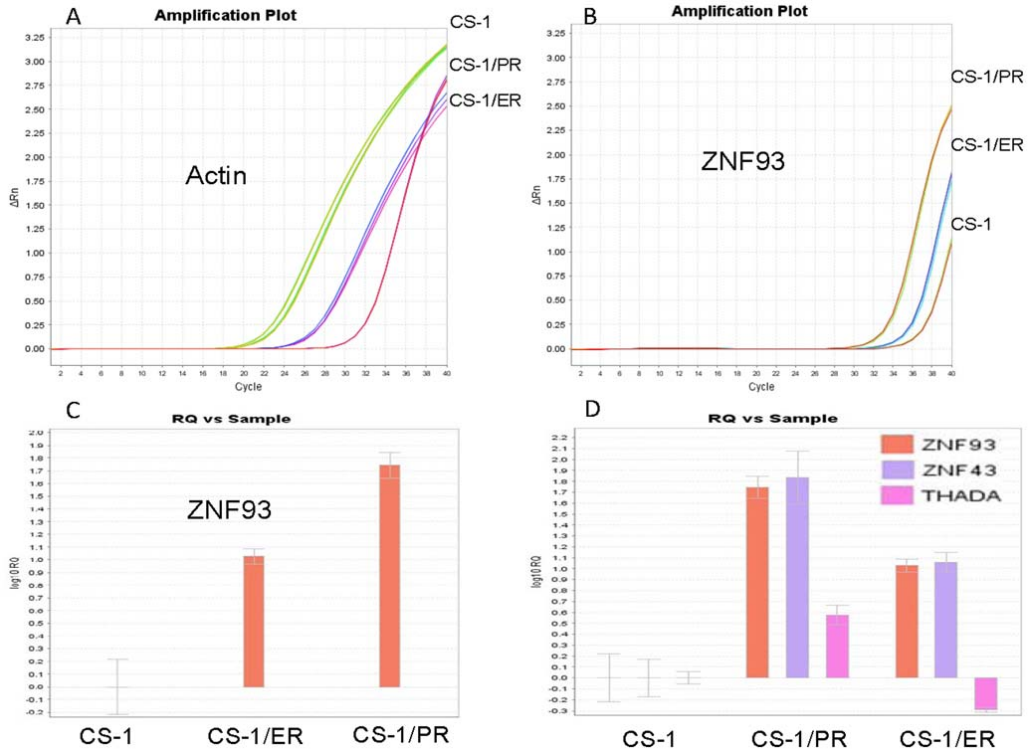
TaqMan real-time RT-PCR evaluates the differentially expressed genes. *A*: Amplification plot for β-actin gene. *B*: Amplification plot for ZNF93 gene. *C*: Relative expression levels of ZNF93 mRNA. *D*: Summary of relative expression levels of ZNF93, ZNF43 and THADA mRNA.

To validate the array data, we performed Real-Time RT-PCR of three genes that were differentially expressed in the two resistant cell lines. ZNF93, ZNF43, and THADA RNA expression were measured by TaqMan RNA assay kit. Differential expression of ZNF93 and ZNF43 were confirmed in both CS-1/ER and CS-1/PR resistant cell lines (Fig. 3A, Fig. 3B and Fig. 3C). THADA overexpression was not confirmed in resistant cell line CS-1/ER (Fig. 3D).

### Expression of ZNF93 in other multidrug resistant cell lines

**Figure 4:**
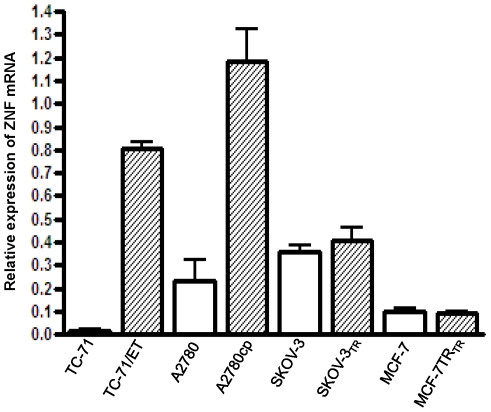
ZNF93 mRNA expression in multiple multi drug resistant cell lines by real-time RT-PCR. All real-time RT-PCR data have been normalized to β-actin.

ZNF93 gene overexpressed in both CS-1/ER and CS-1/PR cell lines and the function of ZNF93 is unclear although the protein has been implicated in the cellular transcriptional regulation. As ZNF93 gene has not previously been linked to drug resistance and was selected for further study. We further evaluated the expression of ZNF93 in other multidrug resistant cell lines by real-time RT-PCR. The results demonstrated that ZNF93 is overexpressed in two ET-743 resistant Ewing sarcoma cell line, TC-ET, as well as in a cisplatin resistant ovarian cancer cell line, A2780cp, as compared with their ET-743 or cisplatin sensitive parental cell lines, but ZNF93 was not overexpressed in paclitaxel resistant cell lines SKOV-3_TR_ and MCF-7_TR_ (Fig. 4).

### Effects of inhibition of ZNF93 expression by siRNA on drug sensitivities

**Figure 5:**
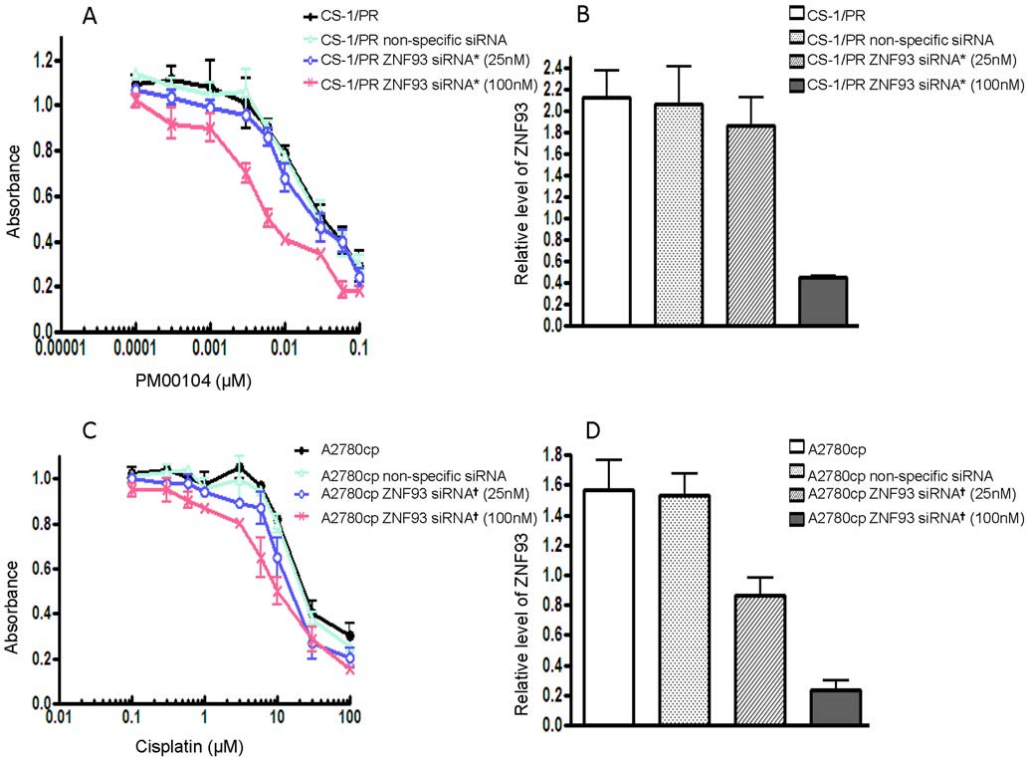
Effect of ZNF93 inhibition on drug sensitivity in CS-1/PR and A2780cp cells. *A*: CS-1/PR was transfected with ZNF93 siRNA*(Applied Biosystems) as well as non-specific siRNA. *C*: A2780cp was transfected with ZNF93 siRNA ^†^ (Dharmacon) as well as non-specific siRNA. The relative sensitivity of each treatment to PM00104 or cisplatin was determined by MTT analysis 72 hours post-transfection . *B and D*: Confirmation of ZNF93 knockdown by real-time RT-PCR. Total RNA was isolated 72 hours post-transfection and ZNF93 expression was analyzed by real-time RT-PCR.

To determine whether ZNF93 plays a role in ET-743 and PM00104 sensitivity, we inhibited its expression in CS-1/PR cells using siRNA knockdown. The relative drug sensitivities were evaluated by comparison of IC_50_ values determined by MTT in siRNA-treated, non-specific siRNA-treated and non-treated control multidrug resistant cell lines. First, cytotoxicity to ET-743 and PM00104 was measured 4 days after transfection of resistant cells with ZNF93 siRNA oligos from Applied Biosystems. We observed that ZNF93 down-regulation did partially recover sensitivity to PM00104 (Fig. 5A) in CS-1/PR cell line. Real time RT-PCR revealed ZNF93 expression was significantly decreased after the cells treated with siRNA (Fig. 5B). Similar results were found in drug resistant cell line CS-1/ER (Data not shown). In addition, for validation of ZNF93 RNAi specificity, the siGenome SMARTpool Human ZNF93 siRNAs were purchased from Dharmacon with the four target sequences of ZNF93 gene and tested in cisplantin resistant cell line A2780cp. The expression ZNF93 in siRNA transfected A2780cp cells was significantly decreased as evaluated by Real-Time RT-PCR (Fig. 5D). MTT assay showed the IC_50_ values of the siRNA treated A2780cp cells were lower as compared with the untreated resistant lines (Fig. 5C), suggesting that ZNF93 siRNA inhibits ZNF93 expression and partially restores the sensitivity of the resistant cell line to cisplatin.

### Effects of overexpression of ZNF93 on ET-743, PM00104 and cisplatin sensitivities

**Figure 6:**
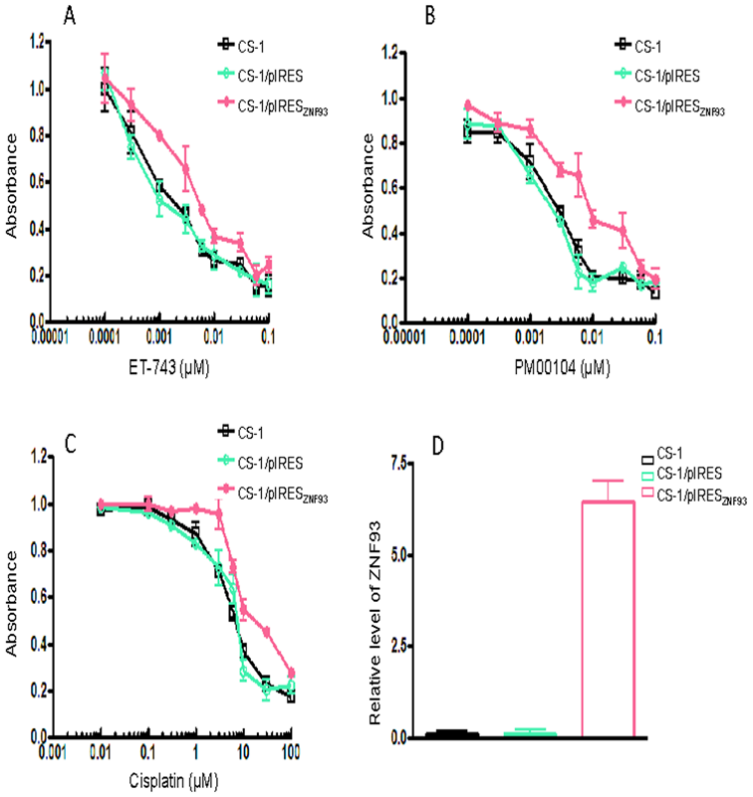
Exogenous expression of ZNF93 confers PM00104, ET743 resistance. *A*, *B* and *C*: Relative cytotoxicity of ET-743,PM00104 and cisplatin in CS-1 derived cell lines (CS-1/pIRES_ZNF93_) stably transfected with a pIRES_ZNF93_ expression vector and in the parental (CS-1) and empty vector (CS-1/pIRES) controls were assessed using the MTT assay. All samples were analyzed in triplicate. *D*: Confirmation of ZNF93 overexpression in pIRES_ZNF93_ tranfected CS-1 cells by real-time RT-PCR as described in Materials and Methods.

Our analyses of endogenous ZNF93 expression demonstrated that ZNF93 is frequently upregulated in ET-743, PM00104 and cisplatin multiple drug resistant cancer cell lines, ZNF93 down-regulation by siRNA could partially recover sensitivity to ET-743,PM00104 and cispatin. These results suggest that the ZNF93 protein may be critically involved in the development of resistance to these drugs. To further determine whether ZNF93 directly participates in the establishment of the resistant phenotype, we transfected the ET-743 and PM00104 sensitive cell line CS-1 with a ZNF93 expression vector and generated stable cell line which overexpress ZNF93 (Figure 6D). We then asked if exogenous overexpression of ZNF93 was sufficient to confer increased ET-743 and PM00104 resistance. The results from the MTT assay in the tranfected cell lines are shown in Fig. 6. ZNF93 transfected CS-1 cells are relatively resistant to ET-743, PM00104 and cisplatin, whereas no significant change in resistance was observed in CS-1 cells transfected with the empty vector (Figure 6A to C). Elevated ZNF93 expression in the tranfected cells was confirmed by real-time RT-PCR (Fig. 6D).

## Discussion

Results from several clinical trials suggest that ET-743 has antitumor activity against several cancer types, including soft tissue sarcomas, breast cancer, and ovarian cancer [2], [3]. Patients with advanced ovarian and breast cancer as well as bone or soft tissue sarcomas typically undergo several lines of anti-cancer regimens before ultimately succumbing to their disease. Multidrug resistance is thought to play an important role in the inevitable failure of tumors to respond to each successive line of chemotherapy. Therefore, understanding the patterns and mechanisms of cross-resistance and finding ways to overcome it is an important goal. Several studies have studied the mechanisms of ET-743 drug resistance with different approaches. However, contradictory results have been published from various groups attempting to correlate resistance to different expression levels of RNAs and proteins in resistant cells [9], [10], [13], [14], [21]. Further resolution of the differences among these results might be best facilitated by genome-wide transcriptional array analysis.

In this study, we report successful establishment of two chondrosarcoma cell lines resistant to ET-743 or PM00104. We then asked how the gene expression levels related to differences in drug resistance in these two cell lines. We used Affymetrix Gene Chip U133 Plus 2 to examine genome-wide expression of RNA transcripts. As compared with several studies using smaller scale gene array, this array completely covers the entire human genome with over 47,000 transcripts. We validated the gene array results for the genes with the most significant changes with real-time RT-PCR. As expected, there are a large number of transcriptional changes associated with *in vitro* acquired resistance to ET-743 and PM00104. Indeed, about 5% to 10% (with cut off value of 2 fold) of transcripts are over-expressed or under-expressed in the resistant cell lines CS-1/ER and CS-1/PR as compared to the sensitive parental cell line.

In the list of top 20 over expressing genes for both CS-1/ER and CS-1/PR, the same three zinc finger protein genes, ZNF93, ZNF43 and ZNF568 (Table 2) were all identified. These genes have not been previously associated with drug resistance. Real-time PCR confirmed ZNF93 and ZNF43 are consistently over expressed in these resistant cell lines (Fig. 3). Preliminary evaluation of ZNF93 confirms that its expression is associated with the multidrug-resistant phenotype in additional cell lines. ZNF93 gene expression was also shown to be increased in ET-743 resistant Ewing's sarcoma cell line (TC-ET) and in the cisplatin-resistant ovarian cancer cell line (A2780cp) but not in the paclitaxel resistant cell lines (SKOV-3_TR_) nor in the paclitaxel resistant breast cancer cell line (MCF-7_TR_), suggesting a fundamentally different mechanism for paclitaxel resistance from ET-743 or PM00104 resistance.

Functional analysis of ZNF93 provides some insight into the epigenetics of ET-743 and PM00104 resistance and might be of use in revealing targets for overcoming drug resistance. Inhibition of ZNF93 using siRNA could partially reverse PM00104, ET-743 and cisplatin resistance in CS-1/PR and A2780cp cells (Fig. 5A). Importantly, overexpression of the ZNF93 protein in a PM00104 and ET-743 sensitive chondrosarcoma cell line CS-1 led to a modest level of PM00104, ET-743 and cisplatin resistance implying an important role for ZNF93 in the development of the drug-resistant phenotype. Other zinc finger proteins such as ZNF143 have been shown to be associated with cisplatin resistance, and ZNF143 depletion using siRNA confers cell sensitivity to cisplatin, but not to oxaliplatin, etoposide and vincristine [23], [24]. ZNF143 has been found associated with tumor suppressor gene p73 but not with p53 expression. Interestingly, p73 could stimulate the binding of ZNF143 to cisplatin-modified DNA. Both Rad51 and flap endonuclease-1 are overexpressed in cisplatin-resistant cells and are target genes of ZNF143[23], [24], [25]. These results suggest ZNF93, ZNF143 as well as other zinc finger proteins may be involved uniquely in DNA repair pathway following DNA damage by ET-743, PM00104, and cisplatin. Future studies on identification of potential ZNF93 target genes for DNA repair pathways are needed to fully understand the molecular mechanism of ZNF93 overexpressin in multidrug resistant cells. Despite its potential role in the transcriptional regulation and DNA repair, ZNF93 may have a potential as an novel target for anticancer therapeutic development. There were reports that defects in the DNA mismatch repair pathway, usually associated with increased resistance to methylating agents and cisplatin, did not affect the cytotoxic activity of ET-743. However, ET-743 did show decreased activity (from 2- to 8-fold) in nucleotide excision repair (NER)-deficient cell lines compared to NER-proficient cell lines [26], [27].

It is obvious that cell lines are not fully representative of tumors from patients, but they do have the advantages of reproducibility, availability, and homogeneity in cell lineage. Further studies on tumor samples to correlate the levels of zinc finger protein such as ZNF93 and DNA repair genes with clinical data will further clarify the biochemical roles of these proteins and their potential as therapeutic targets. The observations described here on gene expression in ET-743 and PM00104 resistant chondrosarcoma cell lines may provide the basis for such future studies.

## References

[1] Rinehart KL (2000). Antitumor compounds from tunicates.. Med Res Rev.

[2] Amador ML, Jimeno J, Paz-Ares L, Cortes-Funes H, Hidalgo M (2003). Progress in the development and acquisition of anticancer agents from marine sources.. Ann Oncol.

[3] Valoti G, Nicoletti MI, Pellegrino A, Jimeno J, Hendriks H (1998). Ecteinascidin-743, a new marine natural product with potent antitumor activity on human ovarian carcinoma xenografts.. Clin Cancer Res.

[4] Cuevas C, Francesch A (2009). Development of Yondelis((R)) (trabectedin, ET-743). A semisynthetic process solves the supply problem.. Nat Prod Rep.

[5] Herrero AB, Martin-Castellanos C, Marco E, Gago F, Moreno S (2006). Cross-talk between nucleotide excision and homologous recombination DNA repair pathways in the mechanism of action of antitumor trabectedin.. Cancer Res.

[6] Soares DG, Escargueil AE, Poindessous V, Sarasin A, de Gramont A (2007). Replication and homologous recombination repair regulate DNA double-strand break formation by the antitumor alkylator ecteinascidin 743.. Proc Natl Acad Sci U S A.

[7] Yin J, Aviles P, Lee W, Ly C, Guillen MJ (2005). Development of a liquid chromatography/tandem mass spectrometry assay for the quantification of PM00104, a novel antineoplastic agent, in mouse, rat, dog, and human plasma.. Rapid Commun Mass Spectrom.

[8] Faircloth G, Cuevas C (2006). Kahalalide F and ES285: potent anticancer agents from marine molluscs.. Prog Mol Subcell Biol.

[9] Kanzaki A, Takebayashi Y, Ren XQ, Miyashita H, Mori S (2002). Overcoming multidrug drug resistance in P-glycoprotein/MDR1-overexpressing cell lines by ecteinascidin 743.. Mol Cancer Ther.

[10] Duan Z, Choy E, Jimeno JM, Cuevas CD, Mankin HJ (2008). Diverse cross-resistance phenotype to ET-743 and PM00104 in multi-drug resistant cell lines. Cancer Chemother Pharmacol..

[11] Erba E, Bergamaschi D, Bassano L, Ronzoni S, Di Liberti G (2000). Isolation and characterization of an IGROV-1 human ovarian cancer cell line made resistant to Ecteinascidin-743 (ET-743).. Br J Cancer.

[12] Riedel RF, Larrier N, Dodd L, Kirsch D, Martinez S (2009). The Clinical Management of Chondrosarcoma. Curr Treat Options Oncol..

[13] Marchini S, Marrazzo E, Bonomi R, Chiorino G, Zaffaroni M (2005). Molecular characterisation of two human cancer cell lines selected in vitro for their chemotherapeutic drug resistance to ET-743.. Eur J Cancer.

[14] Shao L, Kasanov J, Hornicek FJ, Morii T, Fondren G (2003). Ecteinascidin-743 drug resistance in sarcoma cells: transcriptional and cellular alterations.. Biochem Pharmacol.

[15] Morioka H, Weissbach L, Vogel T, Nielsen GP, Faircloth GT (2003). Antiangiogenesis treatment combined with chemotherapy produces chondrosarcoma necrosis.. Clin Cancer Res.

[16] Susa M, Morii T, Yabe H, Horiuchi K, Toyama Y (2009). Alendronate Inhibits Growth of High-grade Chondrosarcoma Cells.. Anticancer Res.

[17] Orth P, Weimer A, Kaul G, Kohn D, Cucchiarini M (2008). Analysis of novel nonviral gene transfer systems for gene delivery to cells of the musculoskeletal system.. Mol Biotechnol.

[18] Duan Z, Feller AJ, Penson RT, Chabner BA, Seiden MV (1999). Discovery of differentially expressed genes associated with paclitaxel resistance using cDNA array technology: analysis of interleukin (IL) 6, IL-8, and monocyte chemotactic protein 1 in the paclitaxel-resistant phenotype.. Clin Cancer Res.

[19] Duan Z, Feller AJ, Toh HC, Makastorsis T, Seiden MV (1999). TRAG-3, a novel gene, isolated from a taxol-resistant ovarian carcinoma cell line.. Gene.

[20] Duan Z, Lamendola DE, Duan Y, Yusuf RZ, Seiden MV (2005). Description of paclitaxel resistance-associated genes in ovarian and breast cancer cell lines.. Cancer Chemother Pharmacol.

[21] Manara MC, Perdichizzi S, Serra M, Pierini R, Benini S (2005). The molecular mechanisms responsible for resistance to ET-743 (Trabectidin; Yondelis) in the Ewing's sarcoma cell line, TC-71.. Int J Oncol.

[22] Carmichael J, DeGraff WG, Gazdar AF, Minna JD, Mitchell JB (1987). Evaluation of a tetrazolium-based semiautomated colorimetric assay: assessment of chemosensitivity testing.. Cancer Res.

[23] Wakasugi T, Izumi H, Uchiumi T, Suzuki H, Arao T (2007). ZNF143 interacts with p73 and is involved in cisplatin resistance through the transcriptional regulation of DNA repair genes.. Oncogene.

[24] Torigoe T, Izumi H, Ishiguchi H, Yoshida Y, Tanabe M (2005). Cisplatin resistance and transcription factors.. Curr Med Chem Anticancer Agents.

[25] Ishiguchi H, Izumi H, Torigoe T, Yoshida Y, Kubota H (2004). ZNF143 activates gene expression in response to DNA damage and binds to cisplatin-modified DNA.. Int J Cancer.

[26] Damia G, Silvestri S, Carrassa L, Filiberti L, Faircloth GT (2001). Unique pattern of ET-743 activity in different cellular systems with defined deficiencies in DNA-repair pathways.. Int J Cancer.

[27] Lin X, Howell SB (2006). DNA mismatch repair and p53 function are major determinants of the rate of development of cisplatin resistance.. Mol Cancer Ther.

